# Neighborhood Factors as Correlates of Alcohol Use in the N2 Cohort Study of Black Sexual Minority Men and Transgender Women

**DOI:** 10.21203/rs.3.rs-4626549/v1

**Published:** 2024-07-18

**Authors:** Tyrone Moline, Dustin T. Duncan, Justin Knox, Seann Regan, Christina A. Mehranbod, Cho-Hee Shrader, John Schneider, Byoungjun Kim

**Affiliations:** Columbia University Mailman School of Public Health; Columbia University Mailman School of Public Health; Columbia University Mailman School of Public Health; Columbia University Mailman School of Public Health; Columbia University Mailman School of Public Health; Columbia University Mailman School of Public Health; University of Chicago Department of Medicine; New York University Grossman School of Medicine

**Keywords:** Alcohol Outlet Density, Alcohol Use, GPS, Sexual and Gender Minorities, Neighborhood Poverty, Neighborhood Disorder, Health Disparities, Selective Daily Mobility Bias

## Abstract

Sexually minoritized men (SMM), transgender women (TW), and particularly Black SMM and Black TW may be disproportionately impacted by alcohol-related problems. Few studies have empirically examined neighborhood factors that may contribute to alcohol use, specifically among these populations. Using data from the N2 longitudinal cohort study in Chicago, IL, survey data from the second wave of longitudinal assessment (n = 126), and GPS mobility data collected during study enrollment were used to evaluate neighborhood alcohol outlet availability, neighborhood disorder, and neighborhood poverty as correlates for individual alcohol use. Neighborhood exposures were measured using 200-m derived activity space areas, created from GPS data, and with publicly accessible geospatial contextual data. Separate multi-variable quasi-poison regression models tested for association between neighborhood alcohol outlet density (AOD), measured separately for on-premise (e.g. bars) and off-premise consumption outlets (e.g. liquor stores), neighborhood poverty (defined as the percentage of neighborhood areas at 150% or greater of the U.S. poverty line), exposure to vacant buildings, and neighborhood violent crime density. Separate analytical models found no significant effect between alcohol use and on-premise neighborhood AOD (*IRR* = 0.99, *p* = *0.35*), off-premise consumption AOD (*IRR* = *0.92*, *p* = *0.33*), or neighborhood violent crime (*IRR* = 1.00, *p* = 0.65). Vacant buildings (*IRR* = *1.03*, *p* = *0.05*) and levels of neighborhood poverty (*1.05*, *p* = *0.01*) were found to be significantly associated with increased alcohol use. Among this population, opposed to geospatial access, neighborhood measurements indicative of disorder and poverty may have greater influence on shaping alcohol use.

## Introduction

In the United States, alcohol use remains a major public health problem, where an estimated 178,00 deaths are attributable to alcohol use occur each year.^1,2^ Sexually minoritized and gender expansive populations, including populations of sexually minoritized men (SMM) and transgender women (TW), have been reported to experience elevated risks of harm from alcohol use.^3,4^

### Neighborhoods and Alcohol Use

Research shows neighborhood environments can effects on health,^5^ with neighborhood conditions additionally being explored as a risk-factor that may contribute to increased risk of individual alcohol use.^6,7^ Specific neighborhood considerations such as access to alcohol, neighborhood socioeconomic status, and neighborhood disorder have primarily been investigated in previous studies as contextual influences pertinent to influencing alcohol use.^7^

The availability of alcohol within a neighborhood is regularly quantified using the measure of alcohol outlet density (AOD), where the count of alcohol retailers within a specified area is divided by the total area within the chosen spatial unit of analysis.^2^ Neighborhood alcohol availability has garnered increased attention as a potential risk factor for alcohol use because of its modifiable nature through zoning/licensing restrictions that can alter the number of licenses distributed or hours/days of sale permitted.^8^ Approaches to limit AOD have already been recommended by governments and organizations at the local, national, and international level.^8,9^ However to date, an absence of studies among populations of multiply-minoritized Black SMM and TW have specifically examined the influence of AOD on alcohol use. This may be especially alarming, given that in comparison to other areas, historically Black neighborhoods and other historically redlined communities of color have been reported to contain disproportionately elevated concentrations of alcohol retailers.^10^

Neighborhood socioeconomic disadvantage may also contribute to risk of alcohol use beyond the influence of individual socioeconomic status alone.^11^ For example, high levels of neighborhood poverty may limit access to essential services, such as the availability of healthcare.^12^ Neighborhood disorder, which describes the various physical and social challenges often illustrative of decay within communities may also contribute to alcohol use. Neighborhood disorder has previously been reported to be associated with alcohol use in multiple other studies.^13^ Elements of disorder such as crime, litter, vandalism, and the presence of vacant buildings are often used to quantity neighborhood disorder.^13^ Concerning alcohol use, neighborhood disorder may influence increased risk of alcohol use through the mechanism of routine chronic stress, where exposure to conditions of disorder such as crime vandalism or distressed areas may lead to alcohol use, as a coping response.^14,15^ Areas with higher levels of neighborhood disorder may also be indicative of broader reduction in social support, where potentially diminished social connections or a reduction in collective efficacy may be observed,^16^ of which, can potentially effect alcohol use as a result.

Methodological limitations that frequent other epidemiological studies of the neighborhood environment have also challenged research on neighborhoods and alcohol use. For example, reviews on environmental context and alcohol use have discussed the flaws of studies implementing residential-based neighborhood definitions for measuring geographic levels of exposure.^7^ When using residential-based methods to quantify neighborhood exposures, researchers commonly apply geocoded participant home addresses as the basis for determining variations in exposure to the neighborhood features of interest. A growing body of research has showcased the flaws associated with this approach. Individuals are mobile and relevant contextual exposures commonly extend beyond residential locations alone or other arbitrary politically-defined spatial boundaries (e.g. ZIP code tabulation area (ZCTA), neighborhood/community area).^17^ Problems such as the modifiable areal unit problem (MAUP)^18^ and the uncertain geographic context problem (UCGP)^19^ are also applicable in this regard. The MAUP specifies how the method of dividing and analyzing geographic areas may shift the categorization of spatial data, whereby study findings may also be altered dependent upon the chosen geographic unit used within a study’s analysis.^18^ Similarly, the UGCP showcases how the geographic boundaries chosen for examination may not represent the actual range of causally relevant contextual environments to which individuals are exposed.^19^ Both of these limitations are applicable to research on the neighborhood alcohol environment and have been commented on with respect to AOD research specifically.^20^ Attempts to address these biases can be achieved through the implementation of neighborhood exposure assessments, using tools such as GPS, that deliberately assess mobility and extend beyond the residential setting.^21^

### Using GPS to Assess Exposure to Features of Neighborhoods

Using GPS devices, researchers can create activity space or activity path areas to quantify the “real” area of environmental exposure,^21^ which may be especially important for highly mobile populations, such as Black SMM.^22^ Beyond this, previous studies on AOD specifically have also discussed how individuals can have a greater cumulative exposure to alcohol outlets outside of their residential setting, further justifying the use of non-residential based measurements when studying neighborhood exposures and alcohol use.^23^

GPS studies are not without methodological challenges, however. The selective daily mobility bias - similar to the neighborhood self-selection bias in non-GPS environmental exposure studies – describes how individuals may voluntarily elect to travel to or frequent areas with greater levels of the environmental conditions under examination.^24^ This phenomenon may impede causal assessments by distorting the true relationship between exposure to environmental conditions and the health outcomes of interest. To illustrate with respect to alcohol use, people with a propensity to drink will likely aggregate, either by traveling to, or choosing to live within, areas with a greater abundance of alcohol retail options.^8^ By incorporating a variable indicating neighborhood preference into study analyses, researchers can attempt to control for this measurement bias. GPS studies controlling for selective daily mobility bias and their challenges to causal inference have been commented on with respect to studies of other health outcomes, such as neighborhood walkability,^25^ but have yet to be addressed within the literature on neighborhoods and alcohol use.

As such, this study uses GPS technology to assess how neighborhood factors, specifically, neighborhood alcohol availability, neighborhood disadvantage, and neighborhood disorder, affect alcohol use in a sample of Black sexual minority men SMM and TW.

## Methods

### Data Collection.

This analysis utilizes data collected from the Neighborhoods and Networks Cohort Study (N2).^26^ Originally launched in Chicago in 2018, the N2 Cohort Study is an ongoing longitudinal cohort developed to investigate social and neighborhood factors contributing to persistent HIV disparities among Black SMM and TW.^26^ Recruitment utilized convenience sampling at a community space and peer referral sampling. Inclusion criteria specified that participants were a) Black or African American, b) lived in the Chicago metropolitan statistical area with no imminent plans to move, c) reported having a sexual encounter with a cisgender man or transgender woman in the past year, and d) consented to study procedures, including wearing a GPS device for two weeks after study enrollment. More exhaustive description of the topics, methods, and findings from the N2 Cohort have been reported elsewhere.^26^

Two forms of data collected from the N2 cohort were used for this analysis. The first, includes responses to a survey questionnaire, collected during the baseline assessment, as well as alcohol use outcome data, collected during the following wave of longitudinal data collection. As alcohol use data was only available from the second wave of longitudinal assessment, where HIV prevention outcomes were emphasized, only persons living without HIV are included in this analysis. The second form of data includes individual geospatial mobility data, collected using GPS devices measured over a two-week period immediately following study enrollment. During the GPS sampling period, participants were instructed to carry a GPS device (BT-Q1000XT, QStarz International Co. Ltd., Taipei, Taiwan) with them, at all times permitting. GPS devices transmitted spatial point location data every ten seconds. Following the GPS sampling period, participants returned the GPS devices and completed other study assessments, where they were then given compensation for their participation.^26^ The collected GPS data were then cleaned using a series of processing scripts to remove time point duplicates within a specified fixed interval, and, to construct 200m-activity path polygon buffer areas for each participant from the raw spatiotemporal point data. Publicly available geospatial data was used for all contextual and neighborhood variables.

### Measures

#### Neighborhood Alcohol Availability

Neighborhood alcohol availability was measured both objectively and subjectively. Objective measurements of neighborhood alcohol availability were calculated using AOD, with the number of alcohol retailers overlapping within each participants activity space divided by the total area within their activity space. For each participant, two AOD measurements were calculated using QGIS software, one for on-premise consumption venues (e.g. bars, restaurants), and one for off-premise consumption outlets (e.g. liquor stores) (Fig. 2.).^2^ Geocoded spatial venue data for on-premise^27^ and off-premise^28^ consumption alcohol retailers within the study area of Chicago created by the Chicago Department of Business Affairs & Consumer Protection were downloaded from the City of Chicago’s open access data repository, the Chicago Data Portal. These data were then filtered, excluding duplicates, those missing geocoordinates, and including only venues with active liquor licenses corresponding to the GPS sampling dates between January 2018 - December 2019.

#### Neighborhood Poverty

To assess neighborhood poverty, we measured average activity space percentage poverty using activity path areas overlayed with 2018 U.S. Census Bureau’s American Community Survey poverty status data,^29^ demarcated at the ZCTA and restricted to City of Chicago boundaries (Fig. 2b.). This measure was weighed by total activity space size, with the final measure showing the mean percentage of total activity space area at 150% or greater of the U.S. poverty line.

#### Neighborhood Disorder

Neighborhood disorder was quantified using two separate measurements. The first, used the count of vacant buildings overlapping within participant activity paths (Fig. 2c.) divided by total activity space area, using geocoded contextual data on vacant and abandoned building violations created by the City of Chicago, accessed through the Chicago Data Portal.^30^ Data on vacant buildings were filtered to remove duplicates, including only those that were reported during study dates. The second measure of neighborhood disorder used activity space area violent crime density using publicly available geocoded crime reporting data from the Chicago Police Department,^31^ accessed through the Chicago Data Portal, and filtered to include only reports matching Federal Bureau of Investigation’s Uniform Crime Reporting system’s classification for violent crime, and which occurred during study dates (Fig. 2d.).

#### Alcohol Use

Participants were asked to describe their past month drinking frequency (number of days), quantity (number of standard drinks consumed on a typical day when drinking), and number of past-month binge drinking days (≥ 5 drinks in one session). The ordinal categorical responses were then scored using integer values from a range of 0–4, assigned based upon level of response to each of the three alcohol use questions. The sum of scores from each of the three questions was then enumerated into a single index measure, intended to capture overall alcohol use across three domains of drinking.

#### Covariates

Survey data were used for the following study variables of age, annual income dichotomized to above or below $12,500 (a categorization based upon closest responses to the US Department of Health and Human Services poverty line for the year 2018), ^32^ educational attainment dichotomized to high school or greater, or less than high school, and quiet-neighborhood residential preference dichotomized to important or not-important, and sexual identity dichotomized to “gay or homosexual” or other.

### Statistical Analysis

To account for overdispersion and extra zeros in the alcohol use data, quasi-Poisson regression models were used to test for association between neighborhood factors and individual alcohol use index scores, with incident rate ratios (IRR) and 95% confidence intervals describing point-estimates. Preliminary analyses included descriptive statistics for all study-variables and tests for correlation between activity space measurements using non-parametric Spearmen correlation coefficients. Bivariate quasi-Poisson regression models tested for unadjusted associations between neighborhood factors and individual alcohol use index scores.

**Figure 1:**
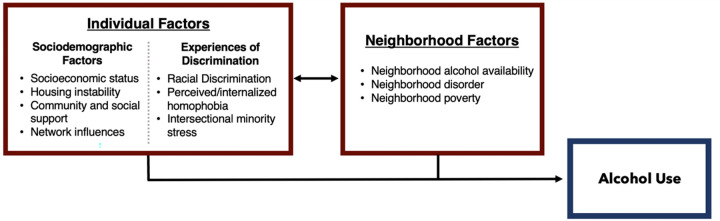
Conceptual diagram depicting individual and neighborhood factors contributing to individual alcohol use, specific to populations of Black SMM and TW, with study neighborhood variables included.

**Figure 2:**
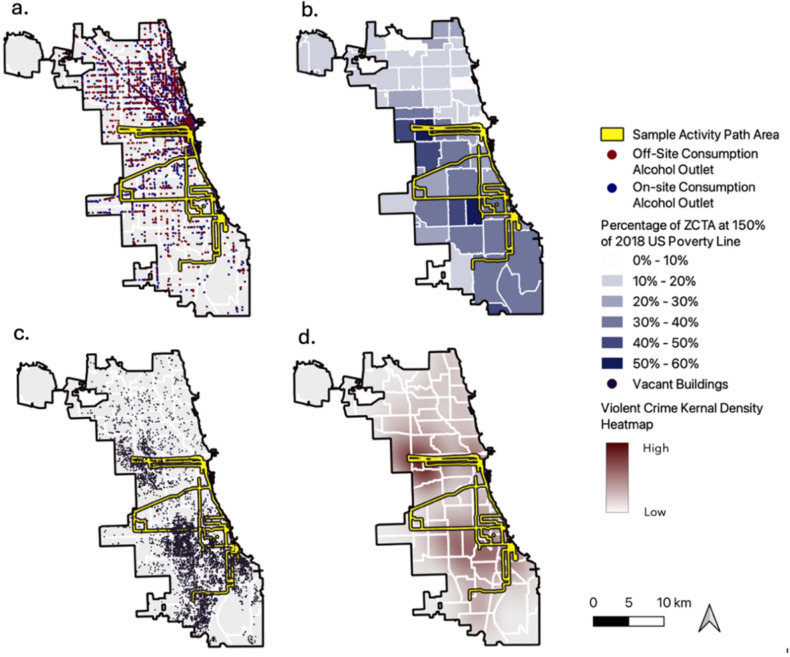
Geographic distribution of neighborhood features examined, with an example 200-m GPS derived activity space area shown in yellow. Data were collected from the N2 Cohort Study participants with alcohol use data (n = 126). a.) neighborhood alcohol availability, with red points indicating off-site consumption alcohol outlets (e.g. liquor stores) and blue points for on-site consumption (e.g. bars) venues. b.) Percentage of ZCTA at 150% of 2018 US poverty line. c.) Location of vacant building complaints. d.) Kernel density heatmap of violent crime location reports, with dark red showing areas of higher violent crime.

Five adjusted quasi-Poisson multivariable regressions then tested separately associations between five neighborhood factors: on-premise AOD, off-premise AOD, violent crime density, vacant building density, and neighborhood poverty and alcohol use. All models included the following covariates of past-three-month housing-instability, age, educational attainment, income, neighborhood preference for quiet neighborhood, and neighborhood poverty, with the exception of the model 3 which considered neighborhood poverty alone. Model parameters and covariates were selected conceptually based upon the hypothesized causal framework (Fig. 1), and upon findings published in similar GPS analyses as seen in other studies among Black SMM and TW.^33^

## Results

Mean participant age was 24.0 years old (Table 1). Ten percent of the sample self-identified as Transgender and 86% of participants reported educational attainment of high school or greater. Slightly more than half the sample indicated past-year annual incomes below $12,500 (51.6%). Further socioeconomic disadvantage was also indicated, as nearly a quarter of the sample (29%) reported experiencing some degree of housing instability within the previous three months (Table 1.). Mean size of GPS activity path areas was 32.4 km ^2^ .

Mean alcohol use index scores were 2.06 (SD = 2.2, Table 1.), with minimum scores of 0 and maximum scores of 11 reported. Of participants, 68% reported any past month alcohol use, with 37% reporting alcohol use on 10 or greater days, and 13% of participant reporting three or more instances of binge drinking within the prior month.

Most participants reported the ease of access to alcohol within their neighborhood as “easy”, with 85% of the sample indicating easy access (Table 1.). Using GPS activity space areas to measure AOD, participants were found to have higher mean levels of AOD exposure to on-premise outlets (10.41 outlets / Km^2^) than to off-premise AOD (2.95 outlets / km2, Table 1.). Mean activity space percentage neighborhood poverty was 34.3% (SD = 5.3). Mean activity space density of vacant buildings was 13.82 buildings/Km^2^, and mean activity space density of violent crimes was 104.25 crimes /Km^2^.

### Bivariate Analyses

Pearson correlation coefficients revealed AOD measurements of different venues types were highly correlated (Supplemental Table 1., *ρ* = 0.956, p < 0.001). AOD measures were also significantly negatively correlated with activity space measurements of neighborhood poverty (Supplemental Table 1, *ρ* = −0.538, p < 0.001; *ρ* = −0.519, p < 0.001) and vacant building density (*ρ* = −0.544, p < 0.001; *ρ* = −0.462, p < 0.001), where those with greater exposure to AOD were also among those with lower activity spaces levels of poverty and with lower exposure to vacant buildings.

Bivariate quasi-Poisson regression models revealed exposure to on-premise AOD approached significant association with lower alcohol use index scores (Table 2. IRR = 0.98, CI: 0.95–1.00, p = 0.06). Greater activity space levels of neighborhood poverty (Table 2. IRR = 1.04, CI: 1.00–1.08, p = 0.03) and vacant building density (IRR = 1.04, CI: 1.01–1.08, p = 0.01) were both significantly associated with increased alcohol use index scores. Additionally, absence of preference for a quiet neighborhood also approached significance on elevating risk of increased alcohol use index scores (p = 0.06).

### Multivariable Analyses

Multivariable quasi-Poisson regression models found neighborhood poverty, measured using average activity space percentage poverty, was significantly associated with higher alcohol use index scores (Table 3. Model 2. IRR = 1.05, CI: 1.02–1.08). However, no evidence of significant effect was found for neighborhood factors quantifying alcohol availability, where levels of activity space exposure to either on-premise AOD (Table 3. Model 1. IRR: 0.99, CI: 0.96–1.01) or off-premise AOD (IRR = 0.91, CI: 0.78–1.06) were not associated with alcohol use. For measures of neighborhood disorder, vacant building density was significantly associated with alcohol use index scores (IRR = 1.03, CI: 1.00–1.06), with no evidence of significant association between activity space violent crime density and alcohol use.

## Discussion

To our knowledge, this is the first study to examine the association between various neighborhood factors with alcohol use among a sample of Black SMM and TW. In this study, we used GPS methods to examine the association between multiple neighborhood factors, such as alcohol availability, neighborhood disorder, and neighborhood disadvantage, and their association with individual alcohol use.

Most participants reported high levels of perceived alcohol availability. However, quantitative measurements of AOD, regardless of outlet venue type examined, were not found to be associated with greater levels of alcohol use. Similarly to what has been reported in other research, even after controlling for individual socioeconomic status and individual mobility preferences, exposure to measures of neighborhood disadvantage and disorder were associated with alcohol use.^34^ In previous studies, disorder is often used as the mechanism to explain how areas of disadvantage contribute to greater alcohol use, where increased exposure to psychosocial stressors and reduced collective efficacy may lead to elevated chronic stress and alcohol use as a response.^14,16^ This study suggests that among Black SMM and TW, neighborhood features of disadvantage and disorder, beyond individual socioeconomic positionality alone may further contribute to risk of alcohol use. This may be an important finding, as approaches investigating neighborhood effects absent dynamic measurements of neighborhood exposures as well as considerations of the differences in the neighborhood socioecological features explored may result in biased conclusions. Future work should continue to differentiate how neighborhood disadvantage and areas of disorder differ, with these two often being analyzed and interpreted as synonymous phenomenon.

Within Chicago, a densely populated urban area where alcohol access is generally very high, there may be insufficient variability in exposure among this limited sample to identify potential associations between AOD and alcohol use. Given the relative socioeconomic disadvantage reported among this group, and the significant influence of other neighborhood factors such as disorder and disadvantage, it is likely other spatial and social considerations are more influential to shaping alcohol use, independent from geospatial access.

In bivariate models unadjusted for variation in socioeconomic status, greater exposure to alcohol outlets approached evidence of a significant protective effect on alcohol use. However, this finding may have been an artifact of where greater mobility within areas of greater socioeconomic advantage and/or lower disorder was protective of alcohol use, rather, than how greater exposure to areas with higher concentrations of outlets was protective for alcohol use.

While this analysis does not report an association between geospatial access and alcohol use, higher levels of neighborhood AOD are likely not without potential harms. Beyond alcohol use, other studies have reported links between neighborhood AOD and increased violence, crime, and risk of injury,^8^ further illustrating the intertwined nature of the study variables examined here.

Another novel finding presented here includes how greater exposure to AOD was found to be significantly correlated with lower levels of exposure to neighborhood features of disorder and/or disadvantage, such as violent crime or density of vacant buildings, of which, differs considerably from what is frequently documented in the literature. Disadvantaged areas are often described to contain higher concentrations of AOD,^10^ however in this study, we did not find evidence of significant relationship between neighborhood AOD and neighborhood measures of disorder or disadvantage. Future research may consider whether disparities in the physical environment of alcohol retailers are a product of the total number of outlets present, or rather, if outlets are elevated relative to the total percentage of retail stores/commercial venues within an area, and/or if this relationship also varies vis-a-vis neighborhood population density.

To our knowledge, we are the first study to both analyze quantitatively the effect of multiple neighborhood features and their effect on alcohol use among a sample of Black SMM and TW, and the first study to consider and control for the effects of the selective daily mobility bias. Other study strengths include the use of GPS to account for individual mobility when quantifying levels of neighborhood exposures.

### Limitations

This study has several limitations. Primarily, while the N2 cohort reports findings from the first cohort of Black SMM and TW assembled to date, the size of the cohort with alcohol use data utilized here remains considerably small and is likely not externally generalizable. Further, measuring alcohol use with self-report surveys may be especially subject to both recall and social desirability bias, where underreporting may have occurred. Second, this analysis utilizes available data on participant alcohol use, but that which was collected during the second longitudinal wave following the GPS sampling period. As exposure-outcome data was not collected simultaneously, alcohol use levels may have also changed. Additionally, because of the availability of geographic data, this analysis was restricted to only include the area within the City of Chicago municipal boundaries. As some participant activity paths extended beyond into the greater Chicagoland surrounding area, potential exposure underestimation may have occurred. This measurement bias may moderate the true effect sizes between neighborhood features examined and alcohol use. While activity space areas can show total areas of individual mobility, the measurements used here do not weight, or place greater emphasis on specific areas where greater time was spent. Additionally, data on violent crime and vacant building locations both relied on community reporting, which may have potentially been underreported relative to their true prevalence, especially within underserved communities. Finally, because of the high correlations between the neighborhood factors examined and due to concerns over multicollinearity, the effect of exposure to each of the neighborhood factors analyzed were only able to be considered separately, and as such, collective effects of multiple exposures simultaneously were unable to be addressed.

### Future Directions

Future research on neighborhoods and alcohol use among Black SMM and TW populations and other populations alike should continue to consider neighborhoods as potential environmental determinants of alcohol use. Many historically Black neighborhoods of the United States, including the South and West Sides of Chicago (where many participants in this research are from) have well-documented histories of social challenges borne out from legacies of racist, and segregation-promoting policies.^35^ Interventions to reduce alcohol use among these two distinct populations should further incorporate the impacts of space and local factors, and how these aspects intersect with broader processes contributing to socioeconomic disadvantage. Among Black SMM and TW, future work may also consider use GPS to analyze the effects of neighborhoods and other substance use outcomes, such as cannabis or polysubstance use.^36^

This study also presents findings that may be applicable for future studies examining the neighborhood environment and its effect onto health outcomes. As highlighted by others,^7^ future work to expand upon the application of GPS could further include temporally sensitive alcohol use event data, using methods such as ecological momentary assessment or other interventional tools, such as geofencing^37^. By explicitly linking places where alcohol use occurs to locations or neighborhoods of interest these approaches may enhance the plentiful but often ambiguous spatial data typically collected in GPS studies. Future work should also continue to investigate how spatial self-selection contributes to the relationship between neighborhoods and health behaviors.

This study also adds that growing body of evidence suggesting vacant buildings can represent a challenge to public health. Findings form previous interventional studies exploring the effect of vacant lot remediation have conveyed promising results,^38^ with these findings having potential to also inform community-level alcohol use interventions. Future studies on alcohol use should further consider the role of vacant lots, of which were not analyzed in this study. Collaborative efforts between urban planners, public health agencies, local community organizations, and governmental authorities alike should continue to remain paramount, recognizing the effects of decisions enacted into the physical and built environments can have the capacity to influence health outcomes.

## Conclusion

This study builds upon the body of previous work attempting to analyze the influence of neighborhood factors and their effect on alcohol use, implementing GPS technology an innovation to do so. Variation in the physical availability of alcohol, as measured by AOD, likely does not contribute to alcohol use in this population due to both a) the already ubiquitous nature of access to alcohol reported by participants in their neighborhoods, and b) the more prominent influences of neighborhood disorder and neighborhood disadvantage. To our knowledge, this is the first study of neighborhood factors and their effect on alcohol use specifically in a population of Black SMM and TW, and to control for the selective daily mobility bias. Future research should continue to use GPS to examine additional contextual correlates of alcohol use in Black SMM and TW and other populations alike.

## Figures and Tables

**Table 1:** Sociodemographic characteristics and descriptive statistics among the N2 Cohort of Black SMM and TW in Chicago (N = 126).

	Frequency (%)
Age	
Mean (SD)	24.00 (3.8)
Sexual Identity	
Gay or Homosexual	67 (53.2%)
Bisexual	42 (33.3%)
Other	17 (13.5%)
Gender Identity	
Transgender	13 (10.3%)
Education	
Less than high school	18 (14.3%)
High school or greater	108 (85.7%)
Income	
Less than $12,500	65 (51.6%)
Greater than $12,500	61 (48.4%)
Past Three-Month Housing Instability	37 (29.4%)
Quiet Neighborhood Residential Preference	105 (80.8%)
Important	104 (82.5%)
Alcohol Use Index Score	
Mean (SD)	2.06 (2.2)
Self-Reported Neighborhood Alcohol Availability	
Easy	107 (84.9%)
Not Easy	19 (15.1%)
GPS Measured Activity Space Characteristics	
Area (Km^2^), Mean (SD)	32.40 (24.1)
On-premise Venue AOD (venues/Km^2^), Mean (SD)	10.41 (7.6)
Off-premise Venue AOD (venues/Km^2^), Mean (SD)	2.95 (1.3)
Neighborhood Poverty (%), Mean (SD)	34.27 (5.3)
Density Violent Crimes (Incidents /km2), Mean (SD)	104.25 (24.9)
Density Vacant Buildings (Buildings / Km2), Mean (SD)	13.82 (7.1)

**Table 2:** Bivariate quasi-Poisson regressions of neighborhood factors and alcohol useindex scores in the N2 cohort study of Black SMM and TW (N = 126)

Parameter	cIRR	95% CI	p-value
On-premise venue AOD	0.98	0.95–1.00	0.06
Off-premise venue AOD	0.87	0.75–1.01	0.08
Vacant building density	1.04	1.01–1.06	0.01*
Violent crime density	1.00	0.99–1.01	0.56
Neighborhood % poverty	1.04	1.00–1.08	0.03*
Quiet neighborhood preference, not important	1.51	0.99–2.32	0.06

* = p < 0.05.

cIRR = crude Incidence Rate Ratio, CI = Confidence Interval. Dichotomized categorical variables with reference variables in parentheses include Income, >$12,500 vs <$12,500 (ref), education (less than high school vs. high school or greater (ref)), Past three-month stable housing (yes vs no (ref)), quiet neighborhood preference (not important vs important (ref)).

**Table 3:** Multivariable quasi-Poisson regression models of social and spatial factorsexamined on alcohol use index scores in the N2 cohort of Black Sexual MinorityMen and Transgender Women (N = 126).

	Parameter	IRR	95% CI	p-value
	Neighborhood Alcohol Availability			
**Model 1.**	On-premise AOD	0.99	0.96–1.01	0.35
**Model 2.**	Off-premise AOD	0.92	0.79–1.08	0.33
	**Neighborhood Disadvantage**				
**Model 3.**	Neighborhood Poverty	1.05	1.02–1.08	0.01*
	**Neighborhood Disorder**				
**Model 4.**	Violent Crime Density	1.00	0.99–1.01	0.65
**Model 5.**	Vacant Building Density	1.03	1.00–1.06	0.05*

* = p < 0.05.

IRR = Incidence Rate Ratio, CI = Confidence Interval. All models were adjusted for the following covariates: income (>$12,500 vs <$12,500 (ref)), education (less than high school vs. high school or greater (ref)), Past three-month stable housing (yes vs no (ref)), sexual identity, quiet neighborhood preference (not important vs important (ref)).

## Abbreviations

SMM: sexual minority men
AOD: alcohol outlet density
ZCTA: ZIP code tabulation area
MAUP: modifiable areal unit problem
UGCP: uncertain geographic context problem
GPS: global positioning system
